# The role of neuroendocrine differentiation in treatment resistance of prostate cancer and intervention strategies

**DOI:** 10.3389/fonc.2025.1743689

**Published:** 2026-01-07

**Authors:** Baozhen Wang, Yanyun Zhang, Wenbo Zhang

**Affiliations:** 1 The Key Laboratory of Experimental Teratology, Ministry of Education and Department of Pathology, School of Basic Medical Sciences, Cheeloo College of Medicine, Shandong University, Jinan, Shandong, China; 2 Department of Party-Masses Work, Zibo Maternal and Child Health Hospital, Zibo, Shandong, China; 3 Department of Pathology, The Second Affiliated Hospital of Zhengzhou University, Zhengzhou, Henan, China

**Keywords:** CRPC, NEPC, neuroendocrine differentiation, prostate cancer, resistance

## Abstract

Prostate cancer is one of the most common malignancies in men, and resistance to conventional treatments is frequently encountered in clinical practice. Among the mechanisms contributing to this resistance, neuroendocrine differentiation (NED) is particularly significant. NED does not only change the basic biological characteristics of cancer cells but also is capable of inducing resistance to endocrine therapy and chemotherapy, which impact the overall prognosis negatively. While NED drives prostate cancer progression and treatment resistance, its pathophysiology and the mechanisms underlying its development are still poorly understood, which restricts the availability of effective clinical interventions. Hence, a detailed study on the molecular pathways that cause NED and the role of this phenomenon in therapy resistance will be needed to improve treatment outcomes in prostate cancer. This review delineates the role of NED in mediating therapy resistance in prostate cancer and evaluates current therapeutic interventions, with the aim of informing the development of new treatment strategies for this malignancy.

## 1 Introduction

The occurrence of resistance to treatment, especially castration-resistant prostate cancer (CRPC), is one of the biggest clinical issues in the management of prostate cancer and has been largely linked to poor prognosis (1, 2). NED constitutes a clinically significant adaptive mechanism underlying treatment resistance, characterized by transdifferentiation of prostate cancer cells exhibiting distinct morphological and functional alterations, typically induced by sustained androgen deprivation therapy (ADT) or similar selective pressures (3, 4). This shift in phenotype results in a marked decrease in tumor sensitivity to standard therapeutic agents (5, 6). Consequently, understanding the molecular underpinnings of NED is essential for devising effective treatment strategies to combat resistance. A particularly aggressive form of NED is treatment-induced neuroendocrine prostate cancer (t-NEPC), which represents about 20% of CRPC cases following extended ADT (7). While changes in the tumor microenvironment are thought to play a role in the development of NED, the exact mechanisms are not fully understood. Interestingly, research has identified similarities between neurodevelopmental processes and the neuroendocrine plasticity induced by therapy during the progression of NED (7), offering new perspectives on prostate cancer resistance and potential strategies for targeting NED. In current clinical practice for metastatic disease, there is an increasing use of second-generation androgen receptor inhibitors and taxane-based chemotherapy as first-line treatments; however, agents like bicalutamide and flutamide continue to be commonly used (8). There is notable variability in treatment responses among patients, with evidence suggesting that a longer duration of CRPC may predict a more durable response to subsequent enzalutamide therapy (9). Consequently, understanding the role of NED in CRPC and its clinical manifestations may provide critical information for optimizing treatment regimens.

The changes in phenotype related to NED are closely connected to significant shifts in the metabolism of tumor cells. This reconfiguration of cellular metabolism is recognized as a key factor in developing resistance to chemotherapy and endocrine therapy, as it not only supports tumor growth but also plays a direct role in making treatments less effective (10, 11). For example, certain changes in how ketone bodies are metabolized in relation to NED have been suggested as a mechanism that leads to drug resistance (12), indicating that this metabolic pathway could be a promising target for future therapies.

In summary, NED plays a pivotal role in the treatment resistance of prostate cancer. Elucidating its molecular mechanisms and functional consequences will significantly impact the development of novel intervention strategies. This article systematically reviews the molecular mechanisms of NED, its contribution to treatment resistance, and potential therapeutic strategies targeting this process, aiming to provide theoretical foundations and practical guidance for clinical management.

## 2 Definition and molecular biomarkers of NED

NED describes a transformation in prostate cancer cells triggered by specific stressors, particularly therapeutic interventions like androgen deprivation therapy or chemotherapy. As a result, these cells adopt neuroendocrine characteristics, such as a decrease in cell size, less cytoplasmic material, and the condensation of nuclear chromatin (13–15). NED exhibits distinct pathological characteristics, most notably poor cellular differentiation, aggressive invasive behavior, and a pronounced propensity for metastatic dissemination. NED is a critical driver of prostate cancer progression, significantly enhancing tumor aggressiveness. The emergence of NED typically indicates disease advancement and is closely linked to poorer clinical outcomes, including an increased risk of metastasis, the development of treatment resistance, accelerated recurrence rates, and reduced overall survival (16–18). These detrimental effects are primarily mediated by disruptions in key signaling pathways and underlying genetic alterations associated with the NED phenotype (19–21).

Molecular biomarkers associated with NED play a vital role in clinical diagnosis and prognostic evaluation. Key neuroendocrine markers include chromogranin A (CgA), neuron-specific enolase (NSE), and synaptophysin (Syn). Elevated levels of these markers serve as characteristic hallmarks of NED, frequently detected in prostate cancer patients and reflective of the neuroendocrine phenotype within tumor cells. Among these, CgA stands as a primary neuroendocrine marker, demonstrating marked elevation across diverse neuroendocrine neoplasms. Critically, its serum/plasma levels exhibit a significant correlation with tumor burden and serve as an indicator of patient prognosis (22). Furthermore, NSE and Syn are frequently employed in conjunction with CgA to monitor the biological behavior of neuroendocrine tumors and assess therapeutic response. Notably, studies demonstrate that NSE exhibits significant diagnostic sensitivity within specific patient cohorts, particularly in cases of small cell lung cancer and other neuroendocrine neoplasms (23, 24).

In recent years, research has increasingly clarified the differences between *de novo* neuroendocrine prostate cancer (*de novo* NEPC) and t-NEPC in terms of molecular characteristics, clinical progression, and treatment response. *De novo* NEPC accounts for approximately 1% of all prostate cancers, often presenting as small cell carcinoma morphology with early loss of genes such as TP53 and RB1, and demonstrating poor response to ADT (20). t-NEPC predominantly occurs in CRPC patients undergoing long-term ADT, with its development closely linked to AR signaling suppression, epigenetic reprogramming, and tumor microenvironment remodeling (7, 25). Therapeutically, *de novo* NEPC exhibits relative sensitivity to platinum-based chemotherapy, whereas t-NEPC often presents with multidrug resistance, potentially requiring targeted interventions against pathways such as PI3K/AKT or EZH2 (26, 27). Consequently, future clinical management should adopt individualized strategies based on the specific NEPC subtype.

## 3 Relationship between NED and treatment resistance in prostate cancer

### 3.1 Role of NED in CRPC

**Figure 1:**
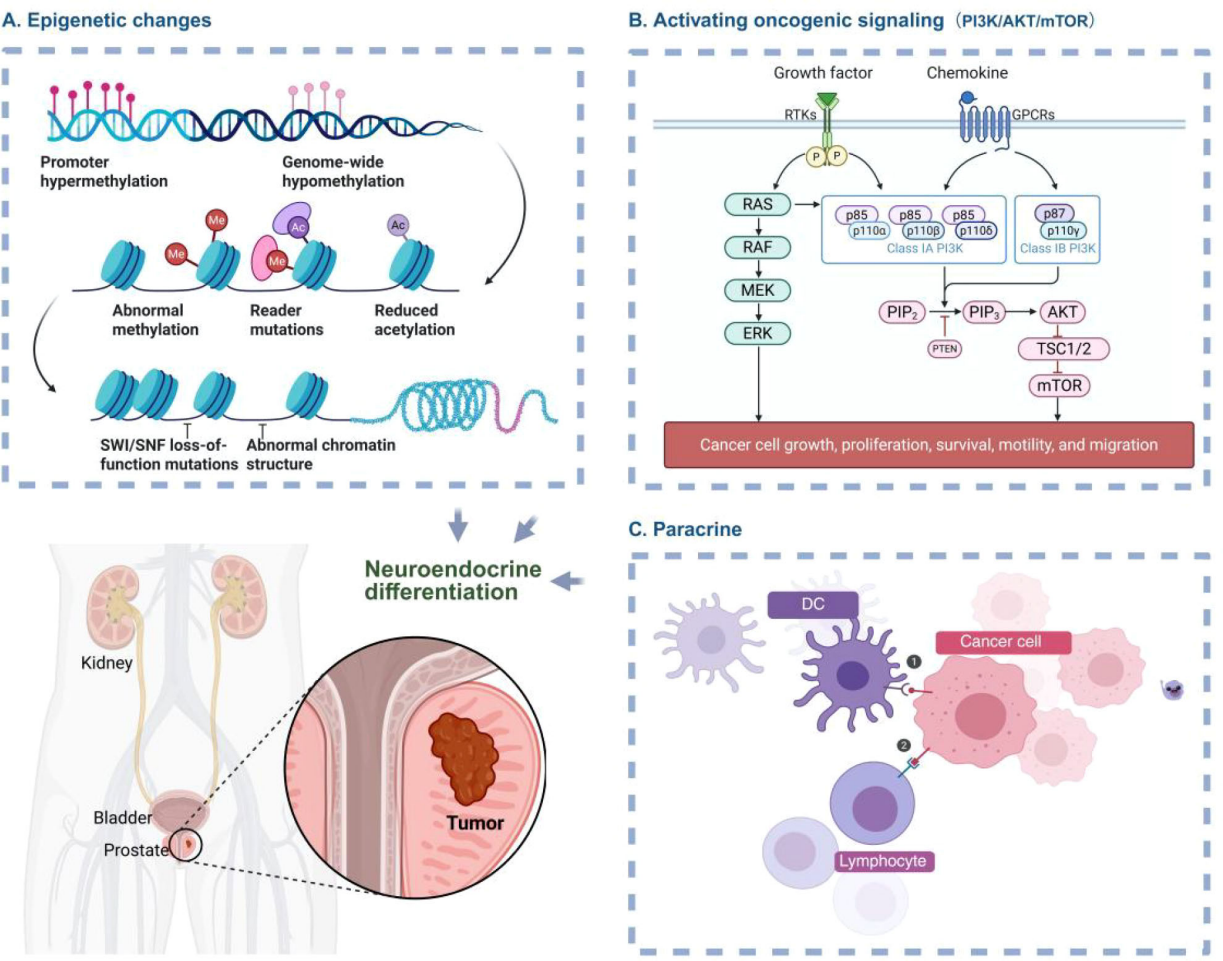
Molecular mechanisms of NED in CRPC. **(A)** NED is closely associated with epigenetic alterations. **(B)** The PI3K/AKT/mTOR pathway modulates NED through regulating cell growth and survival. **(C)** Paracrine mediators from the remodeled tumor microenvironment regulate NED. The figure was created with BioRender.com and is used under a publication license.

NED critically drives the progression of CRPC, particularly in ADT-treated patients (**Figure 1**). This phenotypic metamorphosis is mechanistically linked to adverse clinical outcomes and acquired therapeutic resistance. NEPC develops in approximately 25% of patients with mCRPC undergoing systemic therapy (28). NED cells drive tumor progression through dual mechanisms: intrinsically by altering cellular phenotypes, and extrinsically via paracrine mediators that remodel the tumor microenvironment. Secretion of pleiotropic growth factors and cytokines from NED cells not only enhances adjacent cancer cell proliferation and survival but further compromises therapeutic efficacy (29). This process gives NED cells a survival edge in the tumor environment and leads to resistance against standard hormonal therapies, making patient management and disease progression significantly more challenging (25, 30).

Furthermore, the emergence of NED involves diverse molecular mechanisms, including epigenetic alterations, dysregulated cell cycle control, and inactivation of apoptotic signaling pathways. Collectively, these mechanisms drive CRPC progression, facilitating the transition from androgen-sensitive adenocarcinoma to a more aggressive and therapeutically refractory neuroendocrine phenotype. Researchers are actively investigating targeted intervention strategies against these molecular pathways to develop effective treatments that overcome CRPC resistance and improve patient outcomes (31, 32).

### 3.2 Association of NED with chemotherapy resistance

**Figure 2:**
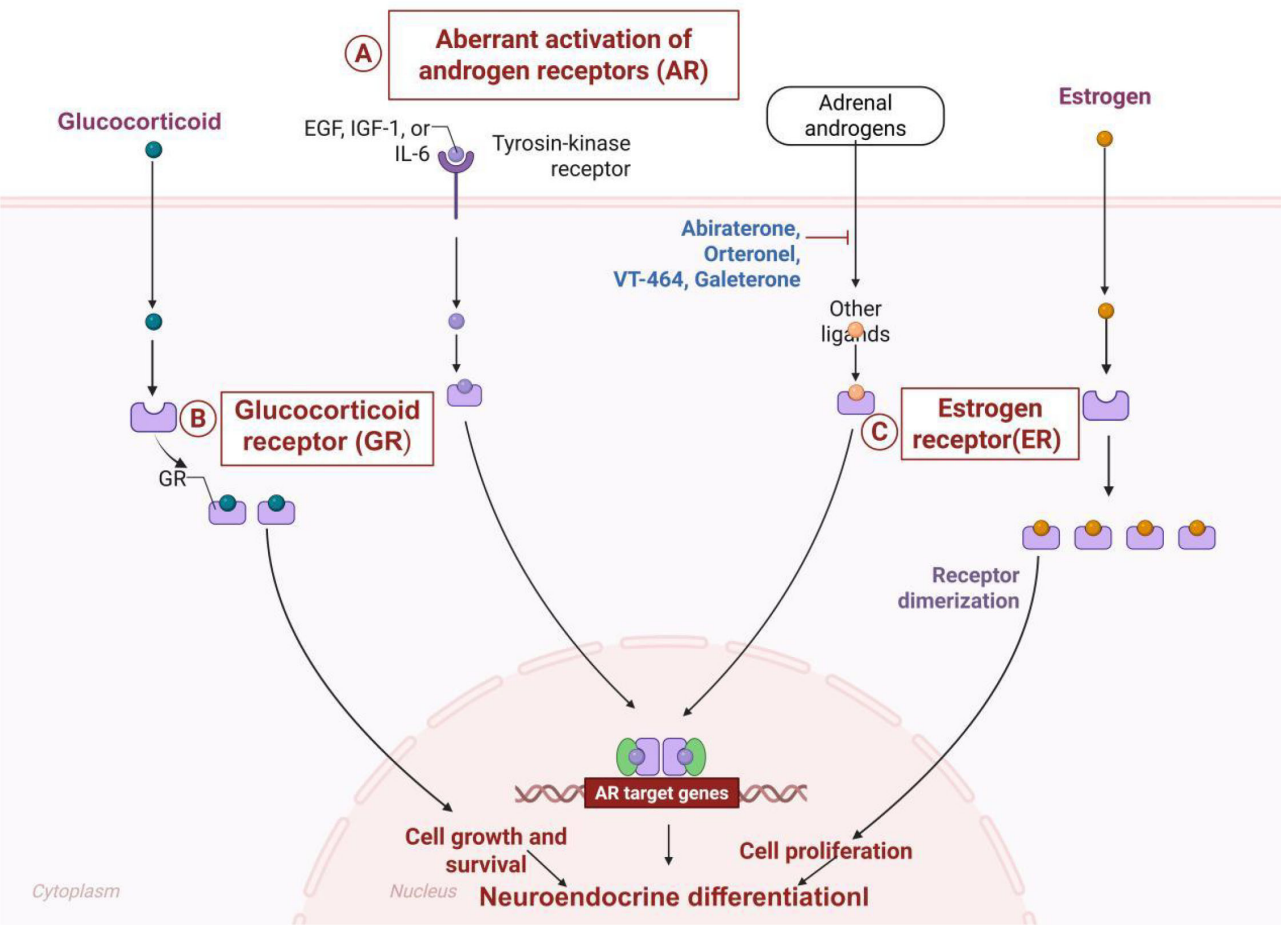
Effects of alterations in hormone signaling pathways. **(A)** AR expression serves as a core driver of NED. **(B)** GR cooperates with NED to drive prostate cancer pathogenesis by promoting tumor cell survival. **(C)** Aberrant ER signaling drives disease progression by promoting cellular transdifferentiation. The figure was created with BioRender.com and is used under a publication license.

NED cells show a marked decrease in sensitivity to standard chemotherapy drugs like docetaxel, and this resistance can be partly explained by irregularities in cell cycle regulation and the ability to avoid programmed cell death, or apoptosis. When exposed to chemotherapy, NED cells often manage to survive the drug-induced cell death, a survival tactic closely tied to their distinct biological characteristics (**Figure 2**). Research indicates that these cells activate certain pro-survival signaling pathways, such as the PI3K/AKT and MAPK pathways, which help them resist the harmful effects of chemotherapy. Importantly, the activation of these pathways is strongly associated with increased growth and survival rates in NED cells (33). Furthermore, the resistance of NED cells to chemotherapy is also affected by changes in their surrounding cellular environment. During chemotherapy, various cytokines and growth factors within the tumor microenvironment promote the survival and proliferation of NED cells. This adaptive alteration of the microenvironment thereby diminishes the efficacy of chemotherapeutic agents. Emerging therapeutic strategies suggest that combined inhibition of key signaling pathways may sensitize NED cells to conventional chemotherapy, potentially improving clinical outcomes (34). This approach highlights the critical need to elucidate the molecular mechanisms underlying NED-mediated chemoresistance, which could inform the development of novel combination therapies for treatment-refractory malignancies.

### 3.3 Implications of NED for immunotherapy resistance

How NED cells evade immune destruction is by the immune system are receiving increasing attention. NED cells frequently exhibit a distinct immunomodulatory profile characterized by downregulation of major histocompatibility complex (MHC) class I molecule expression and concurrent upregulation of immune checkpoint molecules, such as programmed death-ligand 1 (PD-L1). This dual adaptive strategy effectively shields NED cells from immune surveillance, evading detection and destruction by the immune system (34). Specifically, NED cells can impair immune cell function within the tumor microenvironment, thereby diminishing immunotherapy effectiveness. This occurs through the secretion of immunosuppressive cytokines by NED cells, which inhibit T-cell activity and compromise the immune response against tumors (35). Additionally, NED transformation involves alterations in the expression of multiple immune-related genes, potentially contributing to enhanced tumor cell resistance to immunotherapy (36). Consequently, targeting immune evasion mechanisms in NED cells represents a promising therapeutic strategy to enhance immunotherapy responses, achieve better clinical outcomes and improve patient prognosis (37). Such multimodal approaches could provide novel therapeutic alternatives for patients with refractory prostate cancer.

## 4 Molecular mechanisms underlying NED

### 4.1 Alterations in hormonal signaling pathways

NED is critical for prostate cancer progression and treatment resistance. Dysregulation of hormonal signaling pathways is a major driver of NED (**Figure 2**). Prolonged ADT induces decreased androgen receptor (AR) expression and/or AR mutations in prostate cancer cells, with suppression or loss of AR signaling being a central driver of NED (38). Consequently, reduced androgen dependence in these cells fosters their transition to a neuroendocrine phenotype. The further development of NED can be stimulated by aberrant stimulation of other hormonal systems, particularly the glucocorticoid receptor (GR) and estrogen receptor (ER) signaling. Overexpression of the GR stimulates the growth and survival of prostate cancer cells (39), suggesting that GR may be used in conjunction with NED to treat prostate cancer by enhancing the viability of tumor cells. At the same time, cellular transdifferentiation can be induced by aberrant ER signaling, which enables tumor growth and survival (40). The overall dysregulation of these hormonal systems plays a crucial role in modifying intrinsic cancer cell characteristics and reorganizing the tumor microenvironment, which may accelerate the disease’s development. Accordingly, further research on these hormonal changes and their role in NED of prostate cancer is critical to the creation of new treatment methods.

### 4.2 Epigenetic regulation

Epigenetic processes, such as a maladjusted DNA methylation, histone alterations and expression of non-coding RNAs (e.g., miRNAs and lncRNAs), play a crucial role in NED. This association highlights the important role of the epigenetic dysregulation in the pathogenesis of NED (32). As an example, aberrant DNA methylation is capable of silencing tumor suppressor genes thus leading to the development of NEPC. It is worth noting that the downregulation of miR-34a by hypermethylation enhances the differentiation of prostate cancer cells into a neuroendocrine cell type. Moreover, the catalytic subunit of the Polycomb Repressive Complex 2 named as EZH2 is overexpressed and it correlates with the increased tumor aggressiveness and such a phenotypic transformation (26). Moreover, patterns of histone modification are vital in the regulation of genes. High levels of H3K27 acetylation, such as enhance NED by allowing the target genes to be targeted by transcription factors (41). These epigenetic mechanisms contribute to tumor progression by dysregulating core cellular processes, such as proliferation, apoptosis evasion, and the development of treatment resistance. Consequently, therapeutic strategies targeting these epigenetic mechanisms represent a promising approach for novel prostate cancer treatment development.

### 4.3 Activation of cellular signaling pathways

The abnormal reactivation of specific signaling pathways during NED in prostate cancer promotes this phenotypic transition. Notably, the ERK signaling pathway critically mediates neuroendocrine transdifferentiation in these cells. ERK activation promotes tumor cell growth and survival, conferring therapy resistance (42). Additionally, hyperactivation of the PI3K/Akt signaling pathway contributes to the neuroendocrine phenotypic transition in prostate cancer (27, 43). This pathway promotes cancer cell survival via apoptosis inhibition, resulting in enhanced therapy resistance. Elucidating the activation mechanisms and specific functional contributions of these signaling pathways to NED is critical, as it may provide key insights for advancing novel therapeutic strategies. Targeting these pathways represents a promising approach to potentially reverse the NED phenotype and develop innovative treatments for prostate cancer patients.

### 4.4 Role of the tumor microenvironment

The tumor microenvironment (TME) significantly contributes to NEPC development. TMEs linked to NEPC also contain a large number of immune and stromal cells, which release cytokines, such as interleukin-6 (IL-6), that promote tumor growth and metastasis. This intricate cellular and cytokine network fosters a protumorigenic niche, thereby contributing to disease progression and rendering NEPC treatment challenging with therapeutic measures. Recent studies indicate that TME modulation, specifically through cancer therapy using chimeric antigen receptor T-cell (CAR-T), may be a promising mode of therapeutic intervention in NEPC (44, 45). TME composition reconfiguration is a treatment option that is expected to enhance treatment effectiveness and reduce the likelihood of tumor recurrence in NEPC patients. To not only comprehend NEPC biology but also to catalyze the creation of new therapeutic interventions, one must have an extensive understanding of the TME.TMEs linked to NEPC also contain a large number of immune and stromal cells, which release cytokines, such as interleukin-6 (IL-6), that promote tumor growth and metastasis. This intricate cellular and cytokine network fosters a protumorigenic niche, thereby contributing to disease progression and rendering NEPC treatment challenging with therapeutic measures. Recent studies indicate that TME modulation, specifically through cancer therapy using chimeric CAR-T, may be a promising mode of therapeutic intervention in NEPC (44, 45). TME composition reconfiguration is a treatment option that is expected to enhance treatment effectiveness and reduce the likelihood of tumor recurrence in NEPC patients. To not only comprehend NEPC biology but also to catalyze the creation of new therapeutic interventions, one must have an extensive understanding of the TME.

## 5 Clinical detection and diagnosis of NED

### 5.1 Histological examination

NED is one of the main components of the development of various malignancies, especially prostate cancer, and its accurate clinical diagnosis is required. The conventional diagnostic standard for proving NED is immunohistochemistry (IHC), which involves the use of specific antibodies on tumor tissue to define those neuroendocrine markers. Common biomarkers for NED detection include CgA, NSE, and Syn. Among these, CgA and NSE are particularly relevant biomarkers, exhibiting robust expression characteristic of neuroendocrine cells. IHC assessment delineates the spatial distribution and degree of NED, thus guiding critical therapeutic strategies. Advancing research on NED drives the exploration of novel biomarker panels and high-resolution assays, aiming to enhance diagnostic sensitivity and specificity. Current evidence validates that multiplex marker evaluation demonstrates superior diagnostic accuracy for NED, a finding particularly crucial in prostate cancer cohorts. Prostate cancer exemplifies the clinical relevance of NED, where its presence strongly correlates with disease progression and prognostic outcomes (17). This established link underscores the necessity of precise histological quantification for formulating personalized therapeutic strategies and optimizing patient care. Beyond direct clinical utility, histological data constitute an essential reference dataset for clinical investigations evaluating therapeutic impacts on NED phenotypes.

### 5.2 Liquid biopsy technologies

Liquid biopsy technology offers novel, minimally invasive diagnostic modalities for neuroendocrine neoplasms. Both circulating tumor cell (CTC) and circulating tumor DNA (ctDNA) detection have proven indispensable for tumor monitoring and guiding personalized treatment. The characteristics of CTC in real-time describe phenotypic and functional characteristics of disseminated malignancy, hence making it possible to assess the disease and evaluate therapeutic responses. Conversely, ctDNA analysis has established tumor-specific genomic changes and mutations as a substitute for tumor load and its dynamic progressions. A combination of such complementary methods can be used to detect and track NED early and longitudinally, utilizing methods where conventional tissue biopsies are not applicable or yield biased samples. It is important to note that the specific ctDNA signatures are associated with NED, which demonstrates their usefulness as new diagnostic biomarkers. In addition, the longitudinal monitoring of NED status in the context of therapeutic interventions, facilitated by liquid biopsy, enables the timely adjustment of corresponding treatment. Such ability highlights the high clinical promise of liquid biopsy in maximizing the detection and management of NED.

The primary benefit of liquid biopsy is that it enables longitudinal monitoring. In contrast to invasive tissue biopsy, a liquid biopsy can be performed in a non-invasive manner and thus repeated at multiple time points during treatment. This enables the real-time monitoring of tumor progression and response to treatment, providing clinically useful information. Sequential analysis of CTC count and ctDNA dynamics will facilitate longitudinal analysis of non-evidence of disease clinical response and therapy. By way of illustration, in prostate cancer, CTC counts show a high correlation with disease progression, and alterations in CTC levels in the follow-up are an important predictor of cancer progression (46). Moreover, the spectrum and temporal progression of tumor-specific genetic changes are defined via ctDNA profiling, which is important in informing the treatment plan. This non-invasive approach has enabled greater clinical freedom and reduced patient discomfort, thereby significantly improving the dynamic monitoring capability of non-evidence of disease status, which supports the clinical usefulness of liquid biopsy.

### 5.3 Imaging techniques

The initial diagnosis and longitudinal surveillance of neuroendocrine neoplasma would require both conventional and advanced imaging. Positron emission tomography (PET) and magnetic resonance imaging (MRI), in particular, are especially useful in determining the overall tumor burden and helping to identify metastatic dissemination or recurrence of the disease, providing the necessary clinical data. They have been used in prostate cancer, where these imaging modalities provide accurate localization of metastatic lesions and tumor burden, which are essential factors in the clinical management of prostate cancer. PET imaging is the most sensitive method for identifying tumor metabolism activity, while MRI is highly sensitive in terms of soft-tissue contrast and anatomical resolution. The complementary characteristics of these methods make it easier to evaluate the overall status of the disease and burden. Also, therapeutic efficacy will always be accompanied by imaging. A comparative examination of the pre- and post-therapeutic scans helps clinicians assess the patient’s reaction to the treatment and adjust therapeutic operations accordingly. The introduction of new radiotracers, including 68Ga-DOTATATE, has significantly increased the sensitivity and resolution of disease diagnosis, especially in NED cases (47, 48). This radiopharmaceutical is highly selective against somatostatin receptors and specifically optimized to take part in PET imaging in that it allows the localization and anatomical characterization of lesions in NED patients and their systemic distribution. Such receptor-targeted imaging not only significantly elevates the detection threshold for residual disease but also provides critical insights into the underlying molecular and biological features of tumors. Clinical evidence has established that ^68^Ga-DOTATATE PET/CT exhibits high sensitivity for detecting both primary neuroendocrine tumors and their metastatic sites, including early-stage lesions (49). Consequently, this sophisticated imaging modality constitutes a fundamental basis for formulating individualized therapeutic strategies, culminating in improved clinical prognoses. Given the established benefits of novel radiotracers such as ^68^Ga-DOTATATE in the management of NED, they represent a substantial diagnostic advancement and justify expanded clinical adoption (50, 51).

### 5.4 Integrated diagnostic pathway recommendations

Based on current evidence, we propose the following integrated diagnostic and therapeutic strategies (such as surgical relevance) for patients suspected of having NEPC: First, in terms of initial screening, for patients with rapidly progressing CRPC and non-significant PSA elevation, serum CgA and NSE testing is recommended as the first step. Regarding image evaluation, in cases of high clinical suspicion, ^68^Ga-DOTATATE PET/CT is recommended for whole-body assessment of neuroendocrine-active lesions (47–49). Where feasible, perform a needle biopsy of suspicious lesions and conduct immunohistochemical staining for CgA, Syn, and NSE to confirm the diagnosis. Subsequently, analysis of circulating tumor cells (CTCs) and ctDNA can monitor NED-related gene alterations (e.g.TP53, RB1 deletions) to assess treatment response and disease progression during the treatment period (46). This strategy aims to achieve early identification, precise classification, and dynamic monitoring to support clinical decision-making.

### 5.5 Selection of clinical detection methods for NED

In clinical practice, the selection of different diagnostic methods requires comprehensive consideration based on the patient’s specific condition and disease stage. As the gold standard for diagnosing NEPC, tissue biopsy (IHC) is a surgically relevant diagnostic strategy, best suited for cases with clinical suspicion at initial presentation where lesions are amenable to needle aspiration. However, its limitations include invasiveness, potential sampling bias, and the inability to perform dynamic monitoring. Consequently, this method is primarily used to obtain definitive pathological subtyping. Liquid biopsy (CTC/ctDNA) provides a powerful non-invasive tool for longitudinal tumor monitoring. It is particularly suitable for assessing treatment response, tracking clonal evolution, or when tissue samples are unavailable. However, its diagnostic efficacy is highly dependent on the sensitivity and specificity of the detection platform, and it is currently primarily recommended as a means of dynamic monitoring during treatment. Imaging techniques, particularly 68Ga-DOTATATE PET/CT, offer irreplaceable value in the whole-body assessment of neuroendocrine tumor activity, precise staging, and detection of recurrent or metastatic disease. Its limitations include relatively high cost and limited availability. Therefore, this technology is typically recommended for patients with a high clinical suspicion of metastatic or occult NEPC lesions. Additionally, for patients with limited financial resources or those unable to undergo advanced imaging studies, a comprehensive evaluation combining conventional CT/MRI with serum neuroendocrine markers (such as CgA and NSE) is recommended. While ^68^Ga-DOTATATE PET/CT demonstrates high sensitivity (>90%) in diagnosing neuroendocrine tumors, its cost warrants selective use in appropriate patient populations at specialized centers equipped for this modality (49, 50).

## 6 Intervention Strategies Targeting NED

### 6.1 Current treatment options in clinical practice

For patients with CRPC who are highly suspected or confirmed to have NED, the following strategies may serve as clinical references in addition to the traditional platinum-etoposide regimen. Some t-NEPC patients retain residual androgen receptor dependence. Consider switching to a more potent androgen receptor inhibitor (e.g., enzalutamide) or combining with a CYP17 inhibitor (abiraterone) (52). Combination chemotherapy with targeted therapy: Clinical trials combining docetaxel with PI3K/AKT inhibitors have demonstrated combination chemotherapy with targeted therapy have synergistic effects (27, 53). Although PD-1/PD-L1 monotherapy demonstrates limited efficacy, combination approaches with PARP inhibitors or chemotherapy are currently being explored in clinical trials (36). For SSTR-expressing NEPC, ^177^Lu-DOTATATE peptide receptor radionuclide therapy may be considered as an option (18). Clinical decisions should be individualized based on the patient’s physical condition, prior treatment history, biomarker expression, and available medical resources.

### 6.2 Pathway-targeted therapeutics

Agents that target critical signaling pathways offer promising therapeutic options for combating NED. Notable examples of these agents include PI3K/AKT/mTOR inhibitors, such as everolimus, and MAPK inhibitors, like trametinib (54–56). Preclinical studies have shown that these inhibitors can effectively slow down the progression of NED, marking a new approach to treatment. The PI3K/AKT/mTOR pathway plays a crucial role in regulating cell growth, survival, and the transformation of normal cells into cancerous ones across various types of cancer (57). Therefore, inhibiting this pathway can reduce tumor cell growth and prevent the shift to a neuroendocrine phenotype. Likewise, the MAPK pathway is essential for driving oncogenic signaling. Research indicates that these targeted therapies, when used alongside traditional chemotherapy, can work together more effectively, potentially enhancing treatment responses and reducing the likelihood of drug resistance (58, 59). For patients who develop neuroendocrine carcinoma that does not respond to standard treatments, combining these pathway inhibitors with other therapies can provide significant clinical advantages (53, 60).

### 6.3 Epigenetic modulators

Epigenetic therapeutics offer a promising strategy for reversing the molecular changes associated with NED. In particular, inhibitors that target essential epigenetic regulators, such as DNA methyltransferases like decitabine and histone deacetylases like vorinostat, have demonstrated clinical potential in reversing abnormal epigenetic changes, which can help tumor cells regain their normal differentiation (61, 62). While preliminary results from clinical trials indicate some effectiveness in certain patient groups, there is still a need for further research to optimize treatment schedules and identify the best combination strategies. Recent findings suggest that combining epigenetic modulators with traditional chemotherapy or targeted therapies may enhance overall anti-tumor effects (63). Nonetheless, the complex nature of epigenetic regulation highlights the importance of conducting more extensive clinical trials to thoroughly assess long-term safety and the durability of treatment responses (64).

### 6.4 Immunotherapeutic approaches

Immunotherapy applications for NED management are gaining attention despite the limited efficacy observed with immune checkpoint inhibitors (e.g., PD-1/PD-L1 blockers) as monotherapy in NED-dominant malignancies. While response rates to standalone immunotherapies remain modest, combination strategies with conventional treatments may improve clinical responsiveness. Such combinatorial approaches potentially overcome NED-mediated therapeutic resistance through enhanced anti-tumor immune activation. Novel immunotherapeutic modalities-including CAR-T cell therapy-remain in exploratory stages for NED management, though preliminary data indicate potential clinical utility. Current research prioritizes optimizing multimodal regimens integrating these immunotherapies with other treatment modalities to maximize therapeutic efficacy (65, 66).

### 6.5 Emerging therapeutic modalities

Innovative therapeutic avenues are emerging as promising NEPC interventions. Gene therapy approaches and small-molecule inhibitors targeting key pathways (e.g., Notch inhibitors) are undergoing rigorous investigation to identify novel actionable targets (64). Additionally, engineered exosomes, which act as targeted delivery systems for drugs, have demonstrated significant tumor regression in preclinical models of NEPC (67). These innovative approaches not only offer hope for better survival rates but also open up new avenues for research that could lead to clinical applications. As our understanding of NEPC pathobiology deepens, it is expected that this knowledge will pave the way for more personalized and precision-guided treatment strategies in the near future.

### 6.6 Clinical trial progress and prospective treatment strategies

In recent years, multiple clinical trials have been exploring treatment strategies targeting NEPC or NED phenotypes. For example, a Phase II clinical trial evaluated the efficacy of the PARP inhibitor olaparib combined with abiraterone in patients with mCRPC exhibiting neuroendocrine features. Preliminary results indicate that this combination regimen can delay disease progression (52). Additionally, EZH2 inhibitors such as CPI-1205 have demonstrated potential in reversing neuroendocrine phenotypes (26). Moving forward, molecularly stratified “precision immunotherapy-targeted combination therapy” strategies-such as PD-L1 inhibitors combined with AKT inhibitors-are currently in clinical trial design phases. These approaches aim to simultaneously suppress immune evasion and survival signaling in NED cells (27, 36). These advances indicate that future therapeutic strategies should be grounded in surgical oncology, prioritize biomarker-guided combination therapies, and accelerate the clinical translation of NED-specific therapeutic targets.

## 7 Preclinical models and translational research

### 7.1 Cellular models

Genetically modified prostate cancer cell lines (such as LNCaP and PC-3) that undergo NED are crucial tools for elucidating the molecular mechanisms underlying prostate cancer and facilitating drug discovery. Such *in vitro* models are valuable tools that provide a critical platform for studying how NED affects tumor progression and resistance to therapy. For example, it has been found that NED is induced when these cell lines are treated with chemotherapeutic drugs or androgen deprivation therapy, generating a neuroendocrine phenotype similar to that of responsiveness to therapeutic treatments (68). Additionally, these cell lines provide a suitable platform for conducting high-throughput screening assays within the context of identifying novel therapeutics with specific NED inhibition. The standard conditions of such tests allow the profiling and prioritization of potential candidate drugs within a short time (25).

### 7.2 Animal models

Xenograft models, patient-derived xenografts (PDXs), and genetically engineered mouse models (GEMMs) are crucial in the study of NED in living organisms, and they play a vital role in testing novel therapies. These models accurately recapitulate human prostate cancer biology, providing researchers with the opportunity to determine treatment efficacy in domains that most closely simulate clinical procedures. For example, PDX model studies have shown that ADT is associated with the development of NED characteristics, which are known to contribute to treatment resistance (25). In their turn, GEMMs give critical information regarding the molecular pathways involved in NED and cannot be omitted in evaluating the effectiveness of targeted therapies or chemotherapy. Therefore, these *in vivo* models make a significant contribution to the study of NED biology and provide strong experimental models for practical application, enabling the translation of research results into clinical trials.

### 7.3 Organoid models

Organoid models of prostate cancer are gaining relevance as a means of personalized screening of cancer therapy, with models that retain the heterogeneity of patient tumors. These are three-dimensional structures that have been engineered using patient tumor cells and exactly replicate the tumor microenvironment and its interactions with different therapeutic agents. Evidence demonstrates that drug screening performed using patient-derived organoids can reliably predict individual patient treatment responses, thereby advancing the development of personalized therapy. Research indicates that drug screening conducted with patient-derived organoids can reliably predict individual treatment responses, thereby supporting the case for personalized therapy (69, 70). Additionally, organoid models show great potential for NED research, allowing for the assessment of anti-tumor drug effectiveness and the exploration of NED molecular mechanisms within relevant microenvironments (71–73). As methodologies continue to advance, organoid technology is expected to play an increasingly crucial role in future translational research, providing innovative strategies for precision oncology in prostate cancer.

## 8 Prognosis and survival analysis in NEPC

### 8.1 Prognostic factors

NEPC or neuroendocrine prostate cancer exhibits distinct prognostic factors relative to other prostate cancer subtypes. Patients often arrive at the clinic with advanced disease, which is marked by high Gleason scores and specific genetic changes, such as mutations in TP53, RB1, and PTEN. These characteristics are associated with a notably poor survival outlook. In fact, the median overall survival for individuals with NEPC is typically less than 12 months, highlighting the urgent need for prompt treatment (74, 75). Both the advanced stage of the disease at the time of diagnosis and elevated Gleason scores are strongly linked to shorter overall survival and a worse prognosis (76). Identification of these high-risk indicators enables clinicians to initiate earlier therapeutic interventions, potentially improving patient survival outcomes.

### 8.2 Survival data and clinical trials

Clinical trial analyses show that advancements in treating NEPC are still limited, but combination therapies, such as using platinum-based agents alongside etoposide, may offer some modest survival benefits (77, 78). Real-world registry data demonstrate significant heterogeneity in survival outcomes and treatment approaches following NEPC diagnosis, underscoring the complexity of managing this disease (79, 80). Survival analyses indicate that more aggressive treatment strategies during the early stages of the disease can lead to longer survival, especially for patients diagnosed at an earlier stage (81, 82). Therefore, future research should focus on rigorously evaluating the efficacy of multimodal therapies to enable personalized treatment approaches for patients with NEPC.

### 8.3 Patient management and supportive care

Given the rapid disease progression characteristic of NEPC, patients require specialized supportive care and systematic palliative symptom management. The substantial symptom burden throughout the disease trajectory necessitates timely palliative interventions, which significantly enhance quality of life (QoL) (83). Contemporary clinical guidelines underscore the implementation of multidisciplinary approaches-integrating urology, oncology, pain management, and psychological support teams-to deliver comprehensive care and alleviate disease-related distress (84). Evidence confirms that precision-tailored symptom control contributes substantially to maintaining psychological well-being and optimizing overall health status, thus constituting a pivotal element for QoL optimization in NEPC (84).

## 9 Future research directions and challenges

### 9.1 Elucidating molecular mechanisms

NED plays a significant role in treatment resistance in prostate cancer, yet the molecular drivers behind it are not fully understood. Future studies need to identify the driver genes and key signaling pathways associated with NED to discover new therapeutic targets. For instance, the upregulation of TRIM59 has been linked to NED induced by ADT and is associated with shorter survival rates (85). Advanced techniques such as single-cell sequencing and spatial transcriptomics provide valuable insights into the complexity of tumors and the specific roles different cell types play in NED. By combining these innovative technologies, researchers can systematically explore the molecular foundations of NED, paving the way for the development of targeted therapies.

### 9.2 Optimizing clinical trials

Current clinical trials focusing on NED are still limited, highlighting the need for rigorously designed studies to validate effective intervention strategies. The development and validation of biomarkers, such as monitoring NED status, are essential for the success of these trials, especially given that NED in patients with mCRPC is associated with poor outcomes (52, 86). It is crucial for clinical protocols to include assessments through computed tomography imaging and dynamic monitoring of biomarkers to facilitate therapeutic optimization and enhance survival rates. Future trials should emphasize these integrated biomarker approaches to ensure more effective treatment strategies.

### 9.3 Advancing multidisciplinary collaboration

Progress in NED research necessitates the integration of expertise from various fields, including oncology, pathology, molecular biology, and radiology. Establishing collaborative frameworks enables researchers to integrate genomic and transcriptomic datasets, facilitating deeper mechanistic insights and the development of precision therapeutic strategies (87, 88). Collaborative research frameworks foster knowledge sharing, accelerate translational advancements, and optimize clinical management approaches. Therefore, the establishment of dedicated collaborative platforms is crucial for achieving transformative advancements in the care of prostate cancer.

## 10 Conclusions

NED constitutes a pivotal mechanism underlying treatment resistance in prostate cancer, encompassing diverse molecular pathways that are integral to disease progression and the formulation of therapeutic strategies. In addition, recent studies have shown that NED is correlated with various biomarkers, signaling pathways, and tumor microenvironmental processes, which collectively influence tumor development and treatment outcomes. With systematic research of these variables, researchers are increasingly clarifying the essential role of NED in the pathogenesis of prostate cancer. Modern-day evidence suggests that NED is a heterogeneous phenomenon, not due to the existence of a single cause, but rather as a result of a multifaceted relationship between genetic factors, the tumor microenvironment, and previous exposure to therapeutic agents. The innate heterogeneity of the disease leads to differences in clinical manifestations and varied responses to treatment in patients, thereby posing a significant challenge to clinical management. There is therefore an urgent need to ensure that further studies focus more on how this variability is affected on a molecular basis, which will be central in developing more targeted and effective methods of treatment.

Recent technological advances, particularly in next-generation sequencing, single-cell studies, and gene editing, have significantly enhanced our capacity to gain a clear understanding of the pathogenic pathways of neuroendocrine diseases. Such approaches not only allow for the identification of biomarkers but also enable the unravelling of important crosstalk between tumors and their stromal compartment, which in turn opens the way to the identification of new therapeutic targets. Nevertheless, despite the obvious significance of interdisciplinary cooperation in transferring these findings to clinical practice, there are still significant obstacles in planning clinical trials. Current trial models often fail to adequately consider tumor heterogeneity, particularly genetic and molecular heterogeneity. To address these weaknesses, future studies should focus on patient-specific interventions that integrate multidimensional data, including molecular profiling, clinical history, and patient-reported outcomes, to ultimately enhance the oncologic outcomes and quality of life for patients with NED.

In conclusion, NED is a basic pathway that is involved in mediating therapy resistance in prostate cancer. Innovative mechanistic understanding, along with advanced trial designs and customized treatment models is expected to proceed to generate groundbreaking changes in treatment effectiveness. These advancements have a great potential of improving patient survival and quality of life and hence better disease management in the long term.
